# Combining retinal and choroidal microvascular metrics improves discriminative power for diabetic retinopathy

**DOI:** 10.1136/bjophthalmol-2021-319739

**Published:** 2022-02-09

**Authors:** Bingyao Tan, Nicole-Ann Lim, Rose Tan, Alfred Tau Liang Gan, Jacqueline Chua, Simon Nusinovici, Chui Ming Gemmy Cheung, Usha Chakravarthy, Tien Yin Wong, Leopold Schmetterer, Gavin Tan

**Affiliations:** 1 Singapore Eye Research Institute, Singapore; 2 SERI-NTU Advanced Ocular Engineering (STANCE) Program, Singapore; 3 School of Chemical and Biomedical Engineering, Nanyang Technological University, Singapore; 4 Department of Ophthalmology, Yong Loo Lin School of Medicine, Singapore; 5 Singapore National Eye Centre, Singapore; 6 Academic Clinical Program, Duke-NUS Medical School, Singapore; 7 School of Medicine, Dentistry and Biomedical Sciencens, Queen's University Belfast, Belfast, UK; 8 Department of Clinical Pharmacology, Medical University of Vienna, Vienna, Austria

**Keywords:** imaging, retina, choroid

## Abstract

**Purpose:**

To use optical coherence tomography angiography (OCTA) parameters from both the retinal and choroidal microvasculature to detect the presence and severity of diabetic retinopathy (DR).

**Method:**

This is a cross-sectional case–control study. OCTA parameters from retinal vasculature, fovea avascular zone (FAZ) and choriocapillaris were evaluated from 3×3 mm^2^ fovea-centred scans. Areas under the receiver operating characteristic (ROC) curve were used to compare the discriminative power on the presence of diabetes mellitus (DM), the presence of DR and need for referral: group 1 (no DM vs DM no DR), group 2 (no DR vs any DR) and group 3 (non-proliferative DR (NPDR) vs proliferative DR (PDR)).

**Results:**

35 eyes from 27 participants with no DM and 132 eyes from 75 with DM were included. DR severity was classified into three groups: no DR group (62 eyes), NPDR (51 eyes), PDR (19 eyes). All retinal vascular parameters, FAZ parameters and choriocapillaris parameters were strongly altered with DR stages (p<0.01), except for the deep plexus FAZ area (p=0.619). Choriocapillaris parameters allowed to better discriminate between no DM versus DM no DR group compared with retinal parameters (areas under the ROC curve=0.954 vs 0.821, p=0.006). A classification model including retinal and choroidal microvasculature significantly improved the discrimination between DR and no DR compared with each parameter separately (p=0.029).

**Conclusions:**

Evaluating OCTA parameters from both the retinal and choroidal microvasculature in 3×3 mm scans improves the discrimination of DM and early DR.

## Introduction

Diabetic retinopathy (DR) is a microvascular ocular complication of diabetes mellitus (DM), and a leading cause of blindness in working age population.^1–4^ The diabetes population globally is estimated to reach 366 million in 2030, with 34.6% having DR, and 7% having vision-threatening DR.^5 6^ The current classification and staging systems of DR (eg, modified Airlie House/Early Treatment Diabetic Retinopathy Study (ETDRS)^7^ or International Classification^8^) are largely based on examination of changes in the retinal vasculature since these vessels can be easily observed on ophthalmoscopy and colour fundus photography. However, with a new understanding of the pathophysiology and new imaging technology, these DR classification systems may need updates and revision.^9^

Optical coherence tomography angiography (OCTA) has been used to detect retinal microvascular abnormalities associated with DR, such as enlarged and noncircular foveal avascular zone, capillary dropout and high vessel tortuosity,^10–19^ and is well poised to be added as a tool to aid classification of DR. OCTA has the advantages over traditional imaging modalities such as fluorescein angiography (FA) and indocyanine green angiography (ICGA) by being non-invasive and dye injection free, fast and can resolve vascular plexuses in individual layers.^20 21^

Choroidal vascular changes such as choroidal infarcts have long been described in eyes with DR,^22–25^ but these are infrequently quantified clinically or used to determine the severity of DR. Visualising choriocapillaris with FA and ICGA is difficult because of insufficient optical resolution along with limited depth information.^26 27^ Newer swept-source OCTA (SS-OCTA) systems with 1060 nm wavelength now enable choriocapillaris visualisation by precisely segmenting this monolayer plexus underneath Bruch’s membrane.^28–31^ Using such SS-OCTA systems, the choriocapillaris can be characterised by a dense capillary network interspace by flow deficits (FD), also called flow voids. Recent studies have demonstrated choriocapillaris flow impairment in patients with DM with and without DR using commercial OCTA systems.^11 16 32–35^ Rosen described the FD size and number relationship into a power law distribution, whose parameters are altered in different DR severities.^36^

However, it is unknown if combining measures of the retinal and choroidal microvasculature would increase the discriminative ability of OCTA for DM and DR. The objective of our current study was to (1) evaluate retinal and choroidal microvascular parameters measured using an SS-OCTA system in patients with DM and stages of DR, and (2) to determine whether their combination could better detect the presence and severity of DR.

## Methods

### Study participants

We conducted a cross-sectional study. For this analysis, we compared SS-OCTA measures in three groups to evaluate the discriminative power of the OCTA metrics on the presence of DM, the presence of DR and need for referral: group 1 (no DM vs DM no DR), group 2 (no DR vs any DR) and group 3 (non-proliferative DR (NPDR) vs proliferative DR (PDR)).

The study was performed from April 2018 to July 2019 in a single tertiary eye centre, the Singapore National Eye Center, Singapore. Written informed consent was obtained from all participants. For DM participants, the inclusion criteria were patients aged ≥21 years old with type 2 diabetes >5 years duration, while the non-DM population included patients with no known DM. The severity of DR was assessed using two field fundus photography and the ETDRS DR grading scale.^7^ Exclusion criteria were glaucoma, age-related macular degeneration, significant media opacity or diabetic macula oedema. Inclusion criteria for the control participants were no self-reported history of diabetes and evidence of ocular pathology, including glaucoma, age-related macular degeneration or media opacity. IOL Master700 measured the axial eye length, and eyes longer than 26.5 mm were excluded.

This study followed the Strengthening the Reporting of Observational Studies in Epidemiology (STROBE) reporting guideline.

### Optical coherence tomography angiography

We used a prototype SS-OCTA system (PlexElite 9000, Zeiss Meditec, Dublin, California, USA) with a wavelength scanning laser (λ_c_=1050 nm) as the light source. The system operation speed is dependent on the scanning rate of the swept-source (100 000 Ascan/s), and the axial and lateral resolutions in tissue are 6.3 µm and 20 µm, respectively.

The same trained ophthalmic technician scanned all the participants. A 3×3 mm^2^ scanning protocol centred at the fovea was applied, and each data volume consists of 300 A-scans and 300 B-scans. Each B-scan was repeated four times to generate OCTA images using an optical microangiography algorithm.^37^ Motion-related artefacts were minimised by an integrated line scanning ophthalmoscope eye tracker during data acquisition. A review software (Zeiss Meditec) provided automated segmentation of retinal layers and retinal pigment epithelium (RPE). A manual correction was applied for the improper automatic segmentation and choriocapillaris layers were segmented by a standard protocol developed by Spaide^36^ (between 31 µm and 39 µm underneath RPE). Scans were excluded from further analysis if one or more of the following criteria were met: poor clarity images, weak local signals caused by obstacles such as vitreous floaters, and excessive motion artefacts.

The quantification flowchart is shown in online supplemental figure S1. To quantify the vascular components of the retinal circulation, we manually outlined the area of the foveal avascular zone (FAZ) of the superficial and deep vascular plexus FAZ and obtained FAZ size and perimeter. Two annulus masks (500 µm and 1000 µm) were generated around the superficial FAZ, and one annulus mask (500 µm) was generated around the deep FAZ.^38^ Retinal angiograms were binarised by a global threshold, and the area with perfusion was set to 1 whereas the background was set to 0. Perfusion density in each annulus was calculated as the perfused area per total annulus area. The binarised perfusion map was consequently skeletonised (Matlab function: *bwmorph*) to shrink the vessel diameter down to 1 pixel, and vessel density in each annulus was calculated as the vessel length per total annulus area.

10.1136/bjophthalmol-2021-319739.supp1 Supplementary data

**Figure 1:**
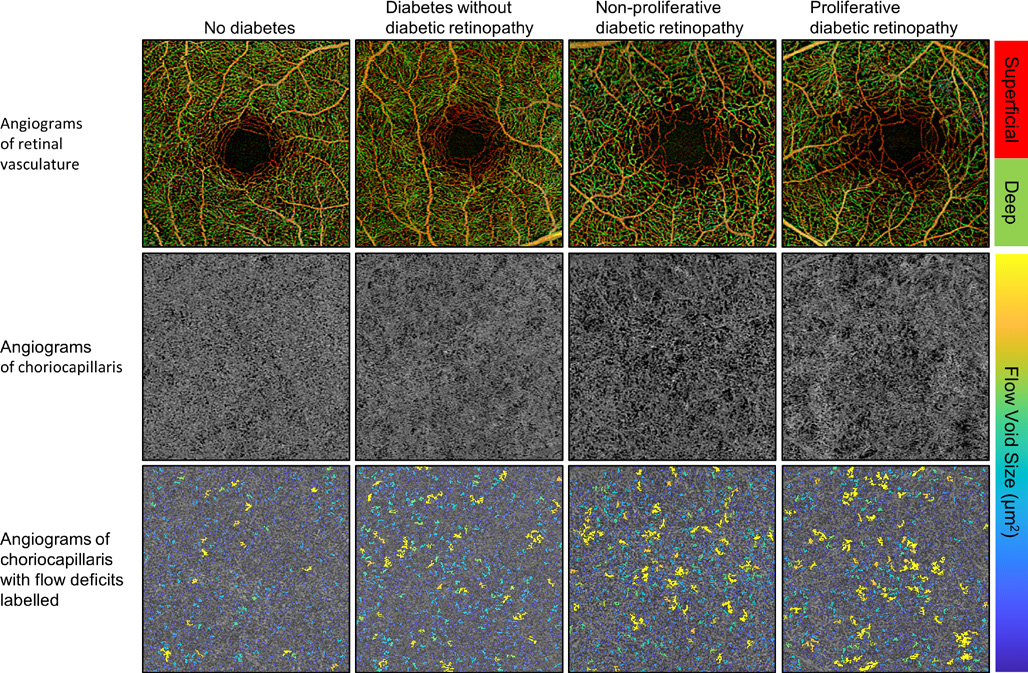
Representative images from different diabetic retinopathy severities.

The extracted choriocapillaris angiograms were first compensated using its corresponding morphological images,^39^ followed by a binarisation using a threshold of mean—SD.^40^ The size (Sz) of each FD was computed, and a size-selective FD density $FDD_{Th}$ was calculated as:

$$FDD_{Th}=\frac{\sum_{i=1}^{N}{Sz}_{i}}{Total\;Imaged\;Area}\left(Sz>Th\right)$$

where N was the number of FDs with size larger than Th, which was set to 200 µm^2^, 400 µm^2^, 600 µm^2^, 800 µm^2^, respectively.^41^ The filter size was decided based on previous reports on high-resolution in-vivo^42^ and ex-vivo^22 43^ choriocapillaris images.

### Statistical analysis

We compared retinal and choroidal microvascular metrics between three groups: group 1 (no DM vs DM no DR), group 2 (no DR vs any DR) and group 3 (NPDR vs PDR). A generalised estimating equation (GEE) was applied to adjust the inter-eye correlation when calculating the association between OCTA metrics and DR severities. Receiver-operating characteristic (ROC) curve analysis was performed to assess the accuracy of each OCTA metric in discriminating different groups and between all classes.^34 44^ GEE cannot be applied in areas under the ROC curve (AUC) where assuming independent observations. CIs and hypothesis tests comparing AUC were calculated using a non-parametric approach to estimate SEs that allowed for correlation of measurements within an individual where both eyes were included. Briefly, bootstrapping was performed with individuals as the resampling units and stratified by DR severity to ensure a representative distribution across the DR severity spectrum in each bootstrap sample.^45^ All OCT metrics were preadjusted for age, gender and systolic blood pressure by using in place of the metrics, the residuals from a linear regression of each metric on these variables. The combined performance of OCT metrics was assessed by simultaneously including them as predictors in a logistic regression model and obtaining model predicted probabilities for each observation.

As the retinal and choriocapillaris metrics, especially five choriocapillaris metrics, were highly correlated with one another, we used principal components analysis to reduce them to fewer components that explain over 90% of the variation in the original metrics. Number of components were selected by inspecting scree plots—two components for choriocapillaris metrics were selected, respectively. These components were included in place of the metrics in a model evaluating the respective diagnostic accuracies of the retinal and choriocapillaris metrics. We reported all AUCs with their 95% CIs and considered p<0.05 a statistically significant improvement in model accuracy.

## Results

### Patient characteristics

A summary of participant characteristics is shown in table 1. The group with no DM with a mean age of 62.1 (6.4) years consisted of 27 participants who contributed a total of 35 eyes. In participants with DM the DR severity was classified into three groups: no DR (62 eyes from 32 patients), NPDR (51 eyes from 29 participants) and PDR (19 eyes from 14 participants). Higher HbA1c levels were associated with DR severity (p=0.001). There was no difference in body mass index (p=0.33), gender (p=0.41), age (p=0.37), diabetes duration (p=0.13), the presence of hypertension (p=0.33), serum glucose (p=0.21), creatinine (p=0.54), cholesterol (p=0.11), high-density lipoprotein (HDL) cholesterol (p=0.52), low-density lipoprotein cholesterol (p=0.05) and triglycerides (p=0.32) among groups, but systolic blood pressure was higher in the PDR group than no DR group (p=0.001), and cholesterol ratio (total/HDL) was higher PDR group than no DR or NPDR (p=0.008).

**Table 1:** Characteristics of study participants by diabetes and DR status

Characteristic	Control (n=27)	No DR (n=32)	NPDR (n=29)	PDR (n=14)	P value
Male, No. (%)	15 (55.6)	22 (68.8)	21 (72.4)	11 (78.6)	0.41
Age, mean (SD), years	62.1±6.4	66.3±6.9	66.2±7.5	64.5±11.7	0.37
BMI	24.5±2.7	25.2±3.6	26.1±4.2	26.1±3.3	0.33
Hypertension, yes (%)	18 (66.7)	26 (81.3)	20 (69)	9 (90)	0.34
Systolic BP (SD), mm Hg	129.6±19.4	147.6±25.6	143.4±20.2	149.4±28.6	0.01*
Diastolic BP (SD), mm Hg	73.0±6.4	75.2±10.6	71.6±9.6	72.0±12.3	0.76
Diabetes duration (SD), years	NA	17.0±8.9	23.7±16.8	20.5±11.4	0.13
Haemoglobin A1c (SD), %	NA	7.0±1.0	8.4±1.7	8.6±1.7	0.001†
Creatinine (SD), µmol/L	NA	87.8±28.4	95.1±40.8	82.3±36.6	0.54
Serum glucose (SD), mmol/L	NA	9.2±4.3	11.0±5.0	11.7±4.1	0.21
Cholesterol (SD), mmol/L	NA	4.2±0.7	4.2±0.9	4.9±0.8	0.11
HDL cholesterol (SD), mmol/L	NA	1.2±0.2	1.2±0.3	1.3±0.4	0.52
Triglycerides (SD), mmol/L	NA	1.9±0.7	2.1±1.2	2.0±0.8	0.32
LDL cholesterol (SD), mmol/L	NA	2.5±0.7	2.4±0.6	2.9±0.8	0.05
Cholesterol ratio (SD)	NA	3.6±1.0	3.6±0.9	3.7±0.8	0.008‡
Axial eye length (SD), mm	NA	24.5±1.1	24.7±1.1	25.0±1.2	0.59

SI conversion factors: to convert cholesterol to mmol/L, multiply values by 0.0259.

Comparison was performed among diabetic groups.

Analysis of variance and post hoc Bonferroni comparing continuous variables and χ^2^ test comparing categorical variables among study groups.

*Significant between control and PDR.

†Significant between no DR and PDR; NPDR and PDR.

‡Significant between no DR and NPDR; no DR and PDR.

BMI, body mass index; BP, blood pressure; DM, diabetes mellitus; DR, diabetic retinopathy; HDL, high-density lipoprotein; LDL, low-density lipoprotein; NPDR, non-proliferative diabetic retinopathy; PDR, proliferative diabetic retinopathy.

Examples of OCTA images from retinal plexuses and choriocapillaris of patients with different DM and DR stages are shown in figure 1. A retinal perfusion reduction in superfical capillary plexus (SCP) and deep capillary plexus (DCP) was associated with severer DR. Worsen DR was also associated with rarefaction of the choriocapillaris and increased number and area of large FDs. The quantitative analysis from retinal and choriocapillaris metrics is summarised in table 2. Boxplots of the choriocapillaris and retinal OCTA metrics further highlighted the relationship between OCTA metrics and DR severities in online supplemental figure S2. As expected, worsening DR was associated with most parameters, but no trend was observed in the deep FAZ area (p=0.619). Interestingly, there was an initial non-significant increase in the DCP in eyes with DM but no DR. Moreover, flow deficit density (FDD) increased with DR severities, and setting a size selectivity on FDD could better stratify the DR severities.

10.1136/bjophthalmol-2021-319739.supp2 Supplementary data

**Figure 2:**
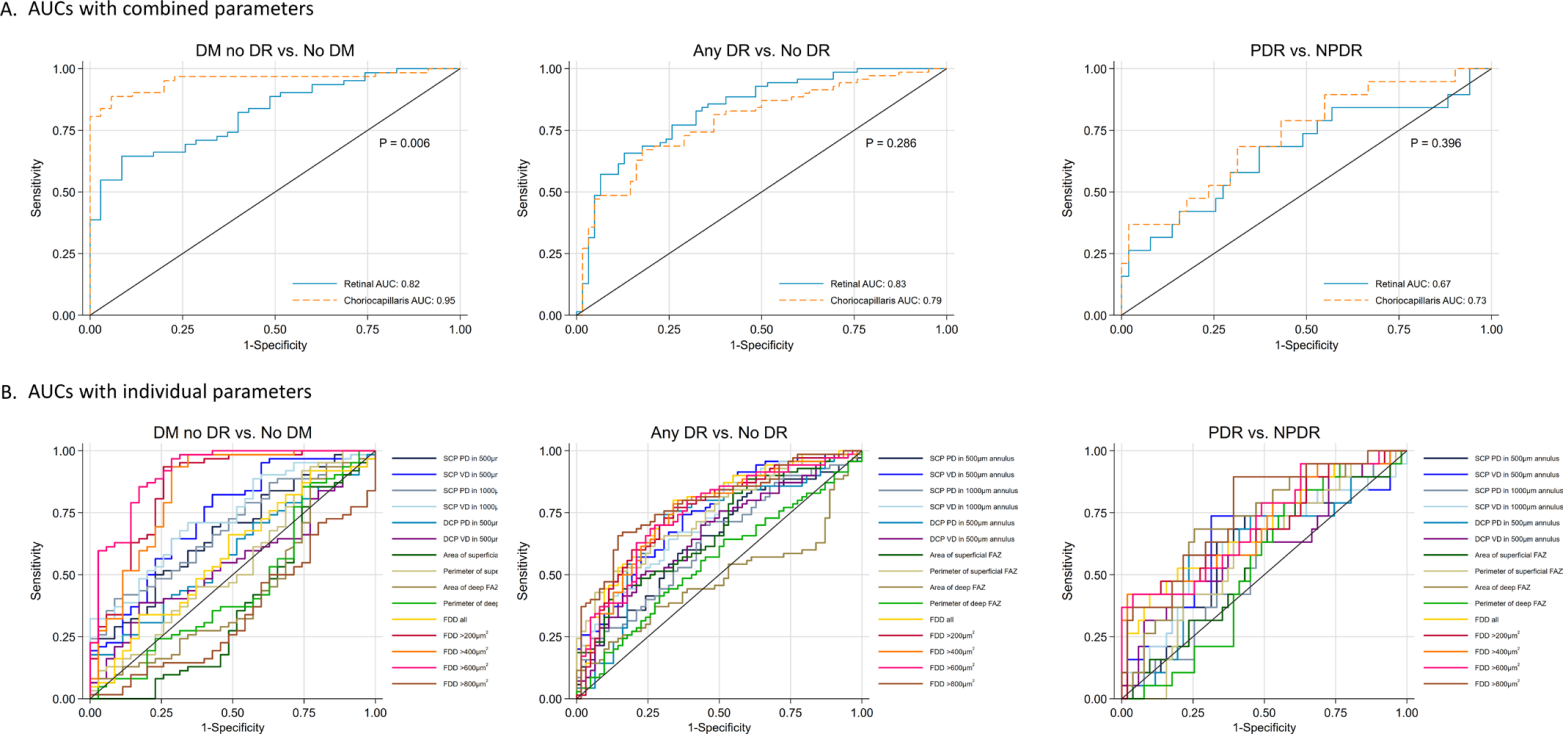
Perfusion density change and discriminative power in stratified DR severities. (A) Total perfusion reduction; (B) capillary reduction; (C) ROC curves for discriminating DR severities using TPD and CPP. AUC, areas under the receiver-operating characteristic curve; DM, diabetes mellitus; DR, diabetic retinopathy; FAZ, fovea avascular zone; FDD, flow deficit density; NPDR, non-proliferative diabetic retinopathy; PDR, proliferative diabetic retinopathy.

**Table 2:** Quantitative metrics from retinal perfusion, FAZ and choriocapillaris

OCTA metrics	No DM	DM no DR	NPDR	PDR	P trend
Retina 500 µm annulus					
SCP perfusion density, %	28.21±1.79	26.24±2.52	25.04±3.29	23.33±4.40	<0.001
DCP perfusion density, %	18.17 (1.82)	18.48 (2.27)	17.11 (2.11)	16.07 (3.69)	0.003
SCP vessel density, %	21.39±1.51	18.48±2.27	17.11±2.12	16.07±3.69	<0.001
DCP vessel density, %	4.61±0.36	4.05±0.56	3.78±0.53	3.48±0.69	0.007
Retina 1000 µm annulus					
SCP perfusion density, %	28.38±1.46	26.47±2.12	25.32±2.72	24.26±4.44	<0.001
SCP vessel density, %	6.18±0.39	5.57±0.57	5.09±0.58	4.68±1.00	<0.001
Superficial FAZ					
Area, mm^2^	0.30±0.13	0.25±0.09	0.32±0.12	0.33±0.10	<0.001
Perimeter, mm	2.16±0.50	2.26±0.46	2.88±0.94	2.97±0.63	<0.001
Deep FAZ					
Area, mm^2^	1.29±0.43	1.25±0.46	1.24±0.70	1.70±0.82	0.619
Perimeter, mm	6.62±1.49	6.42±1.26	6.69±1.70	6.69±1.07	<0.001
Choriocapillaris flow voids density, %					
All	16.08 (0.58)	16.42 (1.05)	17.06 (0.94)	17.63 (1.03)	<0.001
>200 µm^2^	14.43 (0.66)	15.89 (1.02)	16.53 (0.89)	17.17 (1.13)	<0.001
>400 µm^2^	12.13 (0.80)	13.67 (1.02)	14.40 (0.88)	15.35 (1.44)	<0.001
>600 µm^2^	10.24 (0.89)	12.61 (1.08)	13.39 (0.94)	14.46 (1.50)	<0.001
>800 µm^2^	8.74 (0.94)	8.28 (1.43)	9.36 (1.34)	10.77 (1.72)	<0.001

DCP, deep capillary plexus; DM, diabetes mellitus; DR, diabetic retinopathy; FAZ, fovea avascular zone; NPDR, non-proliferative diabetic retinopathy; OCTA, optical coherence tomography angiography; PDR, proliferative diabetic retinopathy; SCP, superficial capillary plexus.

Using both Pearson’s R and R^2^, full correlation matrices are shown in online supplemental figure S3. Parameters from retinal vasculature, FAZ and choriocapillaris were not well correlated (R^2^=0.18 (0.10)), and weak correlations were detected between SCP and DCP parameters (R^2^=0.39 (0.10)), as well as between superficial and deep FAZ parameters (R^2^=0.08 (0.06)).

10.1136/bjophthalmol-2021-319739.supp3 Supplementary data

Further quantification of the diagnostic power of different OCTA metrics was listed in table 3. Among all the retinal OCTA metrics, VD in SCP in its 500 µm annulus had the highest AUC. Moderate discrimination of FAZ metrics was shown in multiple DR severity comparisons. Superficial FAZ perimeters had higher AUC in multiclass comparison (0.691 (0.618 to 0.764)) and DR versus no DR comparison (0.741 (0.662 to 0.820)), while PDR was best discriminated by the area of deep FAZ (0.701 (0.554 to 0.847)) from NPDR.

**Table 3:** Area under the receiver operating characteristic curve for DR severity classification

Parameter	AUC (95% CI)*			
	No DM vs DM no DR group 1	DR vs no DR group 2	PDR vs NPDR group 3	Multiclass†
**Retinal OCTA metrics**				
Retina 500 µm annulus				
SCP perfusion density, %	0.668 (0.530 to 0.805)	0.641 (0.561 to 0.722)	0.613 (0.444 to 0.782)	0.702 (0.626 to 0.778)
DCP perfusion density, %	**0.730 (0.602 to 0.858**)	**0.743 (0.645 to 0.840**)	**0.638 (0.480 to 0.795**)	**0.782 (0.721 to 0.844**)
SCP vessel density, %	0.688 (0.545 to 0.832)	0.633 (0.538 to 0.728)	0.558 (0.400 to 0.716)	0.697 (0.629 to 0.765)
DCP vessel density, %	0.729 (0.596 to 0.863)	0.724 (0.616 to 0.832)	0.617 (0.468 to 0.766)	0.773 (0.709 to 0.837)
Retina 1000 µm annulus				
SCP perfusion density, %	0.577 (0.442 to 0.712)	0.697 (0.611 to 0.783)	0.608 (0.427 to 0.789)	0.702 (0.619 to 0.785)
SCP vessel density, %	0.546 (0.440 to 0.653)	0.663 (0.560 to 0.765)	0.612 (0.415 to 0.809)	0.667 (0.577 to 0.758)
**FAZ metrics**				
Superficial area (mm^2^)	**0.626 (0.482 to 0.770**)	0.678 (0.568 to 0.787)	0.574 (0.430 to 0.717)	0.606 (0.526 to 0.686)
Superficial perimeter (mm)	0.458 (0.315 to 0.600)	**0.741 (0.662 to 0.820**)	0.586 (0.477 to 0.696)	**0.691 (0.618 to 0.764**)
Deep area (mm^2^)	0.568 (0.436 to 0.700)	0.520 (0.441 to 0.599)	**0.701 (0.551 to 0.850**)	0.627 (0.541 to 0.712)
Deep perimeter (mm)	0.546 (0.412 to 0.680)	0.551 (0.464 to 0.638)	0.534 (0.426 to 0.641)	0.533 (0.473 to 0.593)
**Choriocapillaris flow deficits density, %**				
All	0.579 (0.455 to 0.704)	0.768 (0.680 to 0.855)	0.690 (0.515 to 0.866)	0.762 (0.697 to 0.826)
>200 µm^2^	0.853 (0.757 to 0.950)	0.751 (0.665 to 0.837)	0.685 (0.484 to 0.887)	0.834 (0.772 to 0.897)
>400 µm^2^	0.840 (0.729 to 0.950)	0.753 (0.670 to 0.837)	0.699 (0.486 to 0.911)	0.832 (0.771 to 0.893)
>600 µm^2^	**0.912 (0.839 to 0.985**)	0.748 (0.653 to 0.842)	0.709 (0.529 to 0.889)	**0.855 (0.806 to 0.905**)
>800 µm^2^	0.654 (0.504 to 0.804)	**0.788 (0.708 to 0.868**)	**0.755 (0.610 to 0.901**)	0.758 (0.686 to 0.830)
Best one from each category‡	0.927 (0.865 to 0.989)	0.861 (0.800 to 0.922)	0.755 (0.630 to 0.880)	0.878 (0.840 to 0.916)
All parameters included§	0.993 (0.982 to 1.000)	0.907 (0.853 to 0.961)	0.816 (0.698 to 0.934)	0.892 (0.855 to 0.929)
AUC comparison*	z=1.91, p=0.056	z=2.18, p=0.029	z=1.92, p=0.055	z=1.39, p=0.163
**Comparison between retinal and choriocapillaris parameters**				
All retinal parameters	0.821 (0.737 to 0.905)	0.834 (0.753 to 0.916)	0.667 (0.498 to 0.836)	0.838 (0.786 to 0.890)
All choriocapillaris parameters§	0.954 (0.909 to 0.999)	0.789 (0.706 to 0.872)	0.733 (0.577 to 0.889)	0.830 (0.759 to 0.901)
AUC comparison*	z=2.77, p=0.006	z=1.07, p=0.286	z=0.849, p=0.396	z=0.334, p=0.738

The multiclass AUC is calculated as the average of all possible pairwise AUCs, ie, no DR-NPDR and NPDR-PDR.

*CIs and Wald-test p values were calculated using SEs from non-parametric cluster bootstrapping with individuals as the unit of sampling and stratified by DR severity.

†Hand and Till.^67^

‡Parameters selected in bold.

§Adjusted for age, gender and systolic blood pressure.

¶The first two principal components of a principal components analysis of the five flow deficit density parameters.

AUC, areas under the receiver-operating characteristic curve; DCP, deep capillary plexus; DM, diabetes mellitus; DR, diabetic retinopathy; FAZ, fovea avascular zone; NPDR, non-proliferative diabetic retinopathy; OCTA, optical coherence tomography angiography; PDR, proliferative diabetic retinopathy; SCP, superficial capillary plexus.

The discriminative power of choriocapillaris parameters was higher than that of retinal and FAZ parameters for detecting DM and DR. Stratifying choriocapillaris FDD by size could increase the discrimination between DR severities. This difference was significant for group 1 (AUC: 0.579 (0.455 to 0.704) FDD all; 0.912 (0.839 to 0.985) FDD_600_, p<0.001) and multiclass DR detection (AUC: 0.762 (0.697 to 0.826) FDD_600_; 0.855 (0.806 to 0.905) FDD all, p<0.001).

Using a multivariable logistic regression model, AUCs with combined predictors were higher than AUC from any individual predictor. Excellent AUCs were achieved in differentiating groups 1 and 2 and multiclass (AUC >0.89). Comparison between retinal and choriocapillaris parameters in discriminating different DR groups and the performance of individual parameters are shown in figure 2. Retinal and choriocapillaris parameters yielded similar AUC in groups 2 and 3 and multiclass, but group 1 comparison was more accurate to choriocapillaris parameters (p<0.001). Combining all parameters significantly improved the discrimination in group 2 (0.907 (0.853 to 0.961) vs 0.861 (0.800 to 0.922), p=0.029), as compared with selecting the best predictor from each category.

## Discussion

In this study, we evaluated several OCTA metrics known to be affected in type II diabetes in the eyes of persons with and without DR. We examined retinal vascular perfusion density, vessel density, FAZ parameters and choriocapillaris parameters. We also evaluated a multivariable prediction model which combined OCTA parameters from retinal and choroidal microvasculature for DR diagnosis. We found that FDD with a size threshold better stratified DR severity, and our findings suggest that incorporating retinal and choroidal microvascular metrics improves the discriminative power of our models identifying eyes with no DR from those with DR.

Focal and diffuse rarefaction of choriocapillaris has been well documented in patients with diabetes using histology.^22^ OCTA allows quantification of the FDD in vivo with high reproducibility.^41 46^ It has elucidated some of the earliest changes in the retinal vasculature in DR. Notably, focal rarefaction was more prominent in the fovea and is generally due to an excessive change in the size of a few FDs.^22^ Consistent with the previous studies,^10 11 13 14 17 32^ we found that increased FD density was strongly associated with the level of DR, and a significant difference was found between the no DM and DM no DR(p<0.01). The loss of the choriocapillaris or the decrease of the flow signal to the noise floor could result in the merging of the several small FDs into a large FD. This effect would decrease the number of small FD and increase the number of large FD, which could be described as a change of FD size and density relationship.^36 47^ By stratifying FDD by size, and simply calculating the FDD with a size threshold could increase the sensitivity of detecting the rarefaction from both focal and diffuse choriocapillaris degeneration in DR.

Multivariable models perform better than single biomarkers in discriminating most diseases,^48 49^ and in the field of ophthalmology, it has been adopted to predict glaucoma,^50^ myopia^51^ and DR.^52^ Recently, Ashraf *et al* 53 established a multivariable regression model using retinal OCTA metrics to increase the sensitivity and specificity in differentiating NPDR and PDR as well as no DR and no DM. Our study evaluated whether adding OCTA metrics from choriocapillaris will improve discriminatory performance. The retina receives its blood supply from two independent circulations, the retinal and choroidal circulations with different anatomical structures, flow rates and regulatory mechanisms. The retinal vasculature primarily nourishes the inner retina, while the choroidal vasculature mainly provides oxygen and metabolic supply to the outer retina, including photoreceptors.^54^ Compared with retinal vasculature changes, diabetic choroidopathy is less well characterised and its relationship to DR is less clear. The physiological differences between retinal and choroidal circulatory systems in terms of blood flow rate, oxygen saturation rate, haemodynamic properties, autoregulatory function, response to hypoxia^55^ and vessel permeability,^56^ are factors that may explain the lack of correlation between retinal and choroidal blood vascular parameters in the eyes of persons without DM and those with DM. Our data indicate that using the information from both circulatory systems allows better characterisation of the changes in blood flow and vascular regulation that occur with worsening DR. It is notable that in our study we achieved a higher AUC for DR than individual parameter, which is we found to be driven by the parameters that were gained from the analysis of the choriocapillaris. We hypothesise that these OCTA metrices derived through analysis of choriocapillaris might be better biomarkers for early DR.

Other investigators have reported inconsistent findings in the context of OCTA markers in patients with no DRs.^35 57–59^ A recent study by Rosen *et al* reported an initial increase of the PD in patients with DM but no DR using an annulus-based analysis,^58^ while other investigators found no such relationship. The use of the annulus-based analysis can distinguish FAZ, which is affected by ocular magnification.^60 61^ Nonetheless, in keeping with Rosen’s observation,^58^ the PD increased in the DCP but decreased in the SVP. Data from prior work suggest that capillary damage is exacerbated by blood viscosity, decreasing blood cell deformability, platelet aggregation, and increasing blood vessel wall stiffness, which results in capillary closure and retinal ischaemia.^62^ Discrepancy in the parameters observed in the SCP and DCP may indicate the different rates of capillary damage with diabetes in these distinct layers, as tissue hypoxia levels, oxygen extraction rates and haemoglobin oxygen affinity rate are not the same and thereby increasing susceptibility to damage in the DCP.^17 63 64^

Our study has several limitations. We have a relatively small sample size, especially in the PDR group. The image FOV is limited to 3×3 mm which is only a small portion of posterior eye. Patients with pan-retinal photocoagulation (PRP) were not excluded from this study. Although the laser coagulation sites were not within the 3×3 mm FOV, PRP may have a secondary effect on the parafoveal vasculature.^65^ Several patient characteristics were not available in the control group. Our cross-sectional study provided no insight into how well the OCTA detected FD correlate with DR progression. The strict image quality control process excluded 30%–40% of the scans, which might introduce bias in quantification as patients with more severe vision loss may have problems with fixation, which in turn might have resulted in excessive motion artefacts and therefore were more likely to be excluded. The quantification of choriocapillaris can be complicated by instrumental parameters, such as laser power, wavelength, optical resolution, A-scan rate, B-scan rate, sampling rate and motion tracker, as well as algorithm parameters for OCTA calculation, layer segmentation, and FD segmentation. However, discussions on the effect of the parameters on the choriocapillaris quantification are beyond the scope of this paper. The reader is referred to some recent publications that discuss this topic in more detail.^39 40 66^ Finally, we currently do not have an external dataset for the algorithm validation, which is deemed as future work.

In summary, evaluating SS-OCTA parameters from retinal and choroidal microvasculature in 3×3 mm FOV improves the discrimination in early-stage DR, where the predominant changes happen in the choriocapillaris, but not in late-stage DR. It might open on differential therapeutic target sites and potential mechanisms depending on the stage of severity.

## Data Availability

Data are available upon reasonable request.

## References

[1] Flaxman SR , Bourne RRA , Resnikoff S , et al . Global causes of blindness and distance vision impairment 1990-2020: a systematic review and meta-analysis. Lancet Glob Health 2017;5:e1221–34. 10.1016/S2214-109X(17)30393-5 29032195

[2] Cheung N , Mitchell P , Wong TY . Diabetic retinopathy. Lancet 2010;376:124–36. 10.1016/S0140-6736(09)62124-3 20580421

[3] Patel V , Rassam S , Newsom R , et al . Retinal blood flow in diabetic retinopathy. BMJ 1992;305:678–83. 10.1136/bmj.305.6855.678 1393111 PMC1882919

[4] Fong DS , Aiello L , Gardner TW . Retinopathy in diabetes. Diabetes Care 2004. 10.2337/diacare.27.2007.s84 14693935

[5] Ting DSW , Cheung GCM , Wong TY . Diabetic retinopathy: global prevalence, major risk factors, screening practices and public health challenges: a review. Clin Exp Ophthalmol 2016;44:260–77. 10.1111/ceo.12696 26716602

[6] JWY Y , Rogers SL , Kawasaki R . Global prevalence and major risk factors of diabetic retinopathy. Diabetes Care 2012. 10.2337/dc11-1909 PMC3322721 22301125

[7] Treatment E , Retinopathy D . Fluorescein angiographic risk factors for progression of diabetic retinopathy: ETDRS report number 13. Ophthalmology 1991;98:834–40. 10.1016/S0161-6420(13)38015-4 2062516

[8] Wilkinson CP , Ferris FL , Klein RE , et al . Proposed International clinical diabetic retinopathy and diabetic macular edema disease severity scales. Ophthalmology 2003;110:1677–82. 10.1016/S0161-6420(03)00475-5 13129861

[9] Sun JK , Aiello LP , Abràmoff MD , et al . Updating the staging system for diabetic retinal disease. Ophthalmology 2021;128:490–3. 10.1016/j.ophtha.2020.10.008 33218709 PMC8378594

[10] Thompson IA , Durrani AK , Patel S . Optical coherence tomography angiography characteristics in diabetic patients without clinical diabetic retinopathy. Eye 2019;33:648–52. 10.1038/s41433-018-0286-x 30510234 PMC6461750

[11] Carnevali A , Sacconi R , Corbelli E , et al . Optical coherence tomography angiography analysis of retinal vascular plexuses and choriocapillaris in patients with type 1 diabetes without diabetic retinopathy. Acta Diabetol 2017;54:695–702. 10.1007/s00592-017-0996-8 28474119

[12] Mastropasqua R , D'Aloisio R , Di Antonio L , et al . Widefield optical coherence tomography angiography in diabetic retinopathy. Acta Diabetol 2019;56:1293–303. 10.1007/s00592-019-01410-w 31468199

[13] Cao D , Yang D , Huang Z , et al . Optical coherence tomography angiography discerns preclinical diabetic retinopathy in eyes of patients with type 2 diabetes without clinical diabetic retinopathy. Acta Diabetol 2018;55:469–77. 10.1007/s00592-018-1115-1 29453673

[14] Yang J , Wang E , Zhao X , et al . Optical coherence tomography angiography analysis of the choriocapillary layer in treatment-naïve diabetic eyes. Graefes Arch Clin Exp Ophthalmol 2019;257:1393–9. 10.1007/s00417-019-04326-x 31089870

[15] Lupidi M , Cerquaglia A , Gujar R , et al . Functional correlation between choroidal and retinal vascularity in low-grade diabetic retinopathy. Acta Diabetol 2020;57:983–90. 10.1007/s00592-020-01507-7 32201906

[16] Dai Y , Zhou H , Chu Z , et al . Microvascular changes in the choriocapillaris of diabetic patients without retinopathy investigated by swept-source OCT angiography. Invest Ophthalmol Vis Sci 2020;61:50. 10.1167/iovs.61.3.50 PMC7401698 32232345

[17] Nesper PL , Roberts PK , Onishi AC , et al . Quantifying microvascular abnormalities with increasing severity of diabetic retinopathy using optical coherence tomography angiography. Invest Ophthalmol Vis Sci 2017;58:BIO307–15. 10.1167/iovs.17-21787 29059262 PMC5693005

[18] Tan B , Chua J , Lin E , et al . Quantitative microvascular analysis with wide-field optical coherence tomography angiography in eyes with diabetic retinopathy. JAMA Netw Open 2020;3:e1919469. 10.1001/jamanetworkopen.2019.19469 31951275 PMC6991275

[19] Vujosevic S , Toma C , Villani E , et al . Early detection of microvascular changes in patients with diabetes mellitus without and with diabetic retinopathy: comparison between different Swept-Source OCT-A instruments. J Diabetes Res 2019;2019:1–12. 10.1155/2019/2547216 PMC6594252 31281849

[20] Ting DSW , Tan GSW , Agrawal R , et al . Optical coherence tomographic angiography in type 2 diabetes and diabetic retinopathy. JAMA Ophthalmol 2017;135:306–12. 10.1001/jamaophthalmol.2016.5877 28208170

[21] Tsai ASH , Gan ATL , Ting DSW , et al . Diabetic macular ischemia: correlation of retinal vasculature changes by optical coherence tomography angiography and functional deficit. Retina 2020;40:2184–90. 10.1097/IAE.0000000000002721 31842192

[22] Cao J , McLeod S , Merges CA , et al . Choriocapillaris degeneration and related pathologic changes in human diabetic eyes. Arch Ophthalmol 1998;116:589–97. 10.1001/archopht.116.5.589 9596494

[23] Adhi M , Brewer E , Waheed NK , et al . Analysis of morphological features and vascular layers of choroid in diabetic retinopathy using spectral-domain optical coherence tomography. JAMA Ophthalmol 2013;131:1267–74. 10.1001/jamaophthalmol.2013.4321 23907153 PMC4045010

[24] Borrelli E , Sarraf D , Freund KB , et al . OCT angiography and evaluation of the choroid and choroidal vascular disorders. Investig Ophthalmol Vis Sci 2018;58:1–7 10.1016/j.preteyeres.2018.07.002 30059755

[25] Regatieri CV , Branchini L , Carmody J , et al . Choroidal thickness in patients with diabetic retinopathy analyzed by spectral-domain optical coherence tomography. Retina 2012;32:563–8. 10.1097/IAE.0B013E31822F5678 22374157 PMC3393081

[26] Borrelli E , Sarraf D , Freund KB , et al . Oct angiography and evaluation of the choroid and choroidal vascular disorders. Prog Retin Eye Res 2018;67:30–55. 10.1016/j.preteyeres.2018.07.002 30059755

[27] Spaide RF , Fujimoto JG , Waheed NK , et al . Optical coherence tomography angiography. Prog Retin Eye Res 2018;64:1–55. 10.1016/j.preteyeres.2017.11.003 29229445 PMC6404988

[28] Tan B , Chua J , Barathi VA , et al . Quantitative analysis of choriocapillaris in non-human primates using swept-source optical coherence tomography angiography (SS-OCTA). Biomed Opt Express 2019;10:356. 10.1364/BOE.10.000356 30775105 PMC6363185

[29] Chu Z , Gregori G , Rosenfeld PJ , et al . Quantification of Choriocapillaris with optical coherence tomography angiography: a comparison study. Am J Ophthalmol 2019;208:111–23. 10.1016/j.ajo.2019.07.003 31323202 PMC6889046

[30] Choi W , Moult EM , Waheed NK , et al . Ultrahigh-speed, swept-source optical coherence tomography angiography in nonexudative age-related macular degeneration with geographic atrophy. Ophthalmology 2015;122:2532–44. 10.1016/j.ophtha.2015.08.029 26481819 PMC4658257

[31] Migacz JV , Gorczynska I , Azimipour M , et al . Megahertz-rate optical coherence tomography angiography improves the contrast of the choriocapillaris and choroid in human retinal imaging. Biomed Opt Express 2019;10:50. 10.1364/BOE.10.000050 30775082 PMC6363198

[32] Conti FF , Qin VL , Rodrigues EB , et al . Choriocapillaris and retinal vascular plexus density of diabetic eyes using split-spectrum amplitude decorrelation spectral-domain optical coherence tomography angiography. Br J Ophthalmol 2019;103:452–6. 10.1136/bjophthalmol-2018-311903 29793926

[33] Gendelman I , Alibhai AY , Moult EM , et al . Topographic analysis of macular choriocapillaris flow deficits in diabetic retinopathy using swept-source optical coherence tomography angiography. Int J Retina Vitreous 2020;6:1–8. 10.1186/s40942-020-00209-0 32206342 PMC7081691

[34] Conti FF , Song W , Rodrigues EB , et al . Changes in retinal and choriocapillaris density in diabetic patients receiving anti-vascular endothelial growth factor treatment using optical coherence tomography angiography. Int J Retina Vitreous 2019;5:4–11. 10.1186/s40942-019-0192-9 31867124 PMC6902577

[35] Dimitrova G , Chihara E , Takahashi H , et al . Quantitative retinal optical coherence tomography angiography in patients with diabetes without diabetic retinopathy. Invest. Ophthalmol. Vis. Sci. 2017;58:190–6. 10.1167/iovs.16-20531 28114579

[36] Spaide RF . Choriocapillaris flow features follow a power law distribution: implications for characterization and mechanisms of disease progression. Am J Ophthalmol 2016;170:58–67. 10.1016/j.ajo.2016.07.023 27496785

[37] Zhi Z , Qin W , Wang J , et al . 4D optical coherence tomography-based micro-angiography achieved by 1.6-MHz FDML swept source. Opt Lett 2015;40:1779. 10.1364/OL.40.001779 25872072 PMC4612623

[38] Tan B , Sim R , Chua J , et al . Approaches to quantify optical coherence tomography angiography metrics. Ann Transl Med 2020;8:1205. 10.21037/atm-20-3246 33241054 PMC7576021

[39] Tan B , Chua J , Barathi VA , et al . Quantitative analysis of choriocapillaris in non-human primates using swept-source optical coherence tomography angiography (SS-OCTA). Biomed Opt Express 2019;10:356–71. 10.1364/BOE.10.000356 30775105 PMC6363185

[40] Zhang Q , Zheng F , Motulsky EH , et al . A novel strategy for quantifying choriocapillaris flow voids using swept-source OCT angiography. Invest Ophthalmol Vis Sci 2018;59:203–11. 10.1167/iovs.17-22953 29340648 PMC5770182

[41] Lin E , Ke M , Tan B , et al . Are choriocapillaris flow void features robust to diurnal variations? A swept-source optical coherence tomography angiography (OCTA) study. Sci Rep 2020;10:11249. 10.1038/s41598-020-68204-x 32647298 PMC7347889

[42] Kurokawa K , Liu Z , Miller DT . Adaptive optics optical coherence tomography angiography for morphometric analysis of choriocapillaris [Invited]. Biomed Opt Express 2017;8:1803. 10.1364/BOE.8.001803 28663867 PMC5480582

[43] McLeod DS , Lutty GA . High-Resolution histologic analysis of the human choroidal vasculature. Invest Ophthalmol Vis Sci 1994;35:3799–811. 7928177

[44] Obuchowski NA . Nonparametric analysis of clustered ROC curve data. Biometrics 1997;53:567–78. 10.2307/2533958 9192452

[45] Davison AC , Hinkley DV . Bootstrap methods and their application. Cambridge university press, 1997.

[46] Tan B , Chua J , Barathi VA , et al . Quantitative analysis of choriocapillaris in non-human primates using swept-source optical coherence tomography angiography (SS-OCTA). Biomed Opt Express 2019;10:356–71. 10.1364/BOE.10.000356 30775105 PMC6363185

[47] Spaide RF . CHORIOCAPILLARIS signal voids in maternally inherited diabetes and deafness and in pseudoxanthoma elasticum. Retina 2017;37:2008–14. 10.1097/IAE.0000000000001497 28092344

[48] Zou KH , O'Malley AJ , Mauri L . Receiver-operating characteristic analysis for evaluating diagnostic tests and predictive models. Circulation 2007;115:654–7. 10.1161/CIRCULATIONAHA.105.594929 17283280

[49] Park SH , Goo JM , Jo C-H , . Receiver operating characteristic (ROC) curve: practical review for radiologists. Korean J Radiol 2004;5:11. 10.3348/kjr.2004.5.1.11 15064554 PMC2698108

[50] Ocular Hypertension Treatment Study Group, European Glaucoma Prevention Study Group, Gordon MO , et al . Validated prediction model for the development of primary open-angle glaucoma in individuals with ocular hypertension. Ophthalmology 2007;114:10-9. 10.1016/j.ophtha.2006.08.031 17095090 PMC1995665

[51] Tong L , Wong EH , Chan YH , et al . A multiple regression approach to study optical components of myopia in Singapore school children. Ophthalmic Physiol Opt 2002;22:32–7. 10.1046/j.1475-1313.2002.00003.x 11824645

[52] Harris Nwanyanwu K , Talwar N , Gardner TW , et al . Predicting development of proliferative diabetic retinopathy. Diabetes Care 2013;36:1562-8. 10.2337/dc12-0790 23275374 PMC3661803

[53] Ashraf M , Nesper PL , Jampol LM , et al . Statistical model of optical coherence tomography angiography parameters that correlate with severity of diabetic retinopathy. Invest Ophthalmol Vis Sci 2018;59:4292–8. 10.1167/iovs.18-24142 30167660 PMC6110573

[54] Schmetterer L , Kiel JW . Ocular Blood Flow. Springer Science & Business Media, 2012.

[55] Geiser MH , Riva CE , Dorner GT , et al . Response of choroidal blood flow in the foveal region to hyperoxia and hyperoxia-hypercapnia. Curr Eye Res 2000;21:669–76. 10.1076/0271-3683(200008)2121-VFT669 11148604

[56] Stitt AW , Curtis TM , Chen M , et al . The progress in understanding and treatment of diabetic retinopathy. Prog Retin Eye Res 2016;51:156–86. 10.1016/j.preteyeres.2015.08.001 26297071

[57] Simonett JM , Scarinci F , Picconi F , et al . Early microvascular retinal changes in optical coherence tomography angiography in patients with type 1 diabetes mellitus. Acta Ophthalmol 2017;95:e751–5. 10.1111/aos.13404 28211261

[58] Rosen RB , Andrade Romo JS , Krawitz BD , et al . Earliest evidence of preclinical diabetic retinopathy revealed using optical coherence tomography angiography perfused capillary density. Am J Ophthalmol 2019;203:103–15. 10.1016/j.ajo.2019.01.012 30689991 PMC6612596

[59] Al-Sheikh M , Akil H , Pfau M , et al . Swept-source OCT angiography imaging of the foveal avascular zone and macular capillary network density in diabetic retinopathy. Invest Ophthalmol Vis Sci 2016;57:3907. 10.1167/iovs.16-19570 27472076

[60] Sampson DM , Gong P , An D , et al . Axial length variation impacts on superficial retinal vessel density and foveal avascular zone area measurements using optical coherence tomography angiography. Invest Ophthalmol Vis Sci 2017;58:3065. 10.1167/iovs.17-21551 28622398

[61] Llanas S , Linderman RE , Chen FK , et al . Assessing the use of incorrectly scaled optical coherence tomography angiography images in peer-reviewed studies: a systematic review. JAMA Ophthalmol 2020;138:86. 10.1001/jamaophthalmol.2019.4821 31774456

[62] McMillan DE . The effect of diabetes on blood flow properties. Diabetes 1983;32 Suppl 2:56-63. 10.2337/diab.32.2.s56 6400669

[63] Standl E , Kolb HJ . 2,3-Diphosphoglycerate fluctuations in erythrocytes reflecting pronounced blood glucose variation. in-vivo and in-vitro studies in normal, diabetic and hypoglycaemic subjects. Diabetologia 1973;9:461-6. 10.1007/BF00461689 4359285

[64] Duh EJ , Sun JK , Stitt AW . Diabetic retinopathy: current understanding, mechanisms, and treatment strategies. JCI Insight 2017;2. doi:10.1172/jci.insight.93751. [Epub ahead of print: 20 Jul 2017]. PMC5518557 28724805

[65] Cole ED , Novais EA , Louzada RN , et al . Visualization of changes in the choriocapillaris, choroidal vessels, and retinal morphology after focal laser photocoagulation using OCT angiography. Invest Ophthalmol Vis Sci 2016;57:OCT356. 10.1167/iovs.15-18473 27409493

[66] Byon I , Nassisi M , Borrelli E , et al . Impact of slab selection on quantification of Choriocapillaris flow deficits by optical coherence tomography angiography. Am J Ophthalmol 2019;208:397–405. 10.1016/j.ajo.2019.08.026 31493401

[67] Hand DJ , Till RJ . A simple Generalisation of the area under the ROC curve for multiple class classification problems. Mach Learn 2001;45:171–86. 10.1023/A:1010920819831

