# Supplementary figures and images for: Combining retinal and choroidal microvascular metrics improves discriminative power for diabetic retinopathy
Source: Br J Ophthalmol. 2022 Feb 9;107(7):993–9. doi: 10.1136/bjophthalmol-2021-319739 (PMC10359699; doi:10.1136/bjophthalmol-2021-319739)

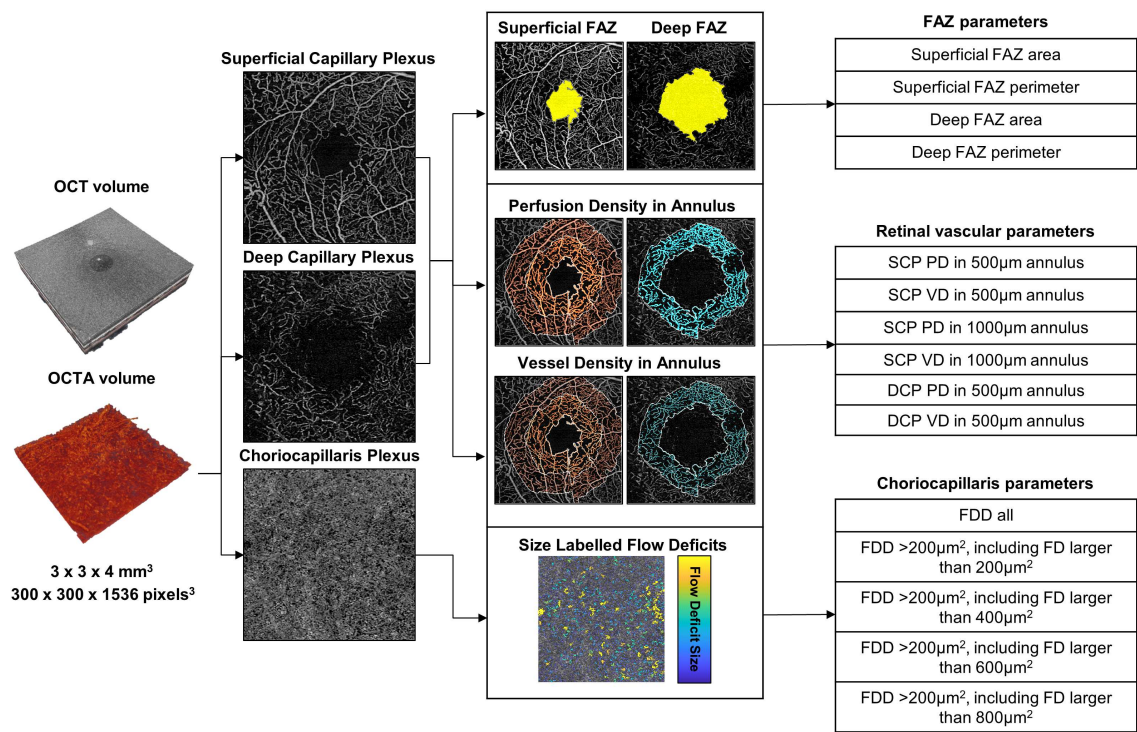

Supplement: Supplementary data [file bjophthalmol-2021-319739supp001.pdf]

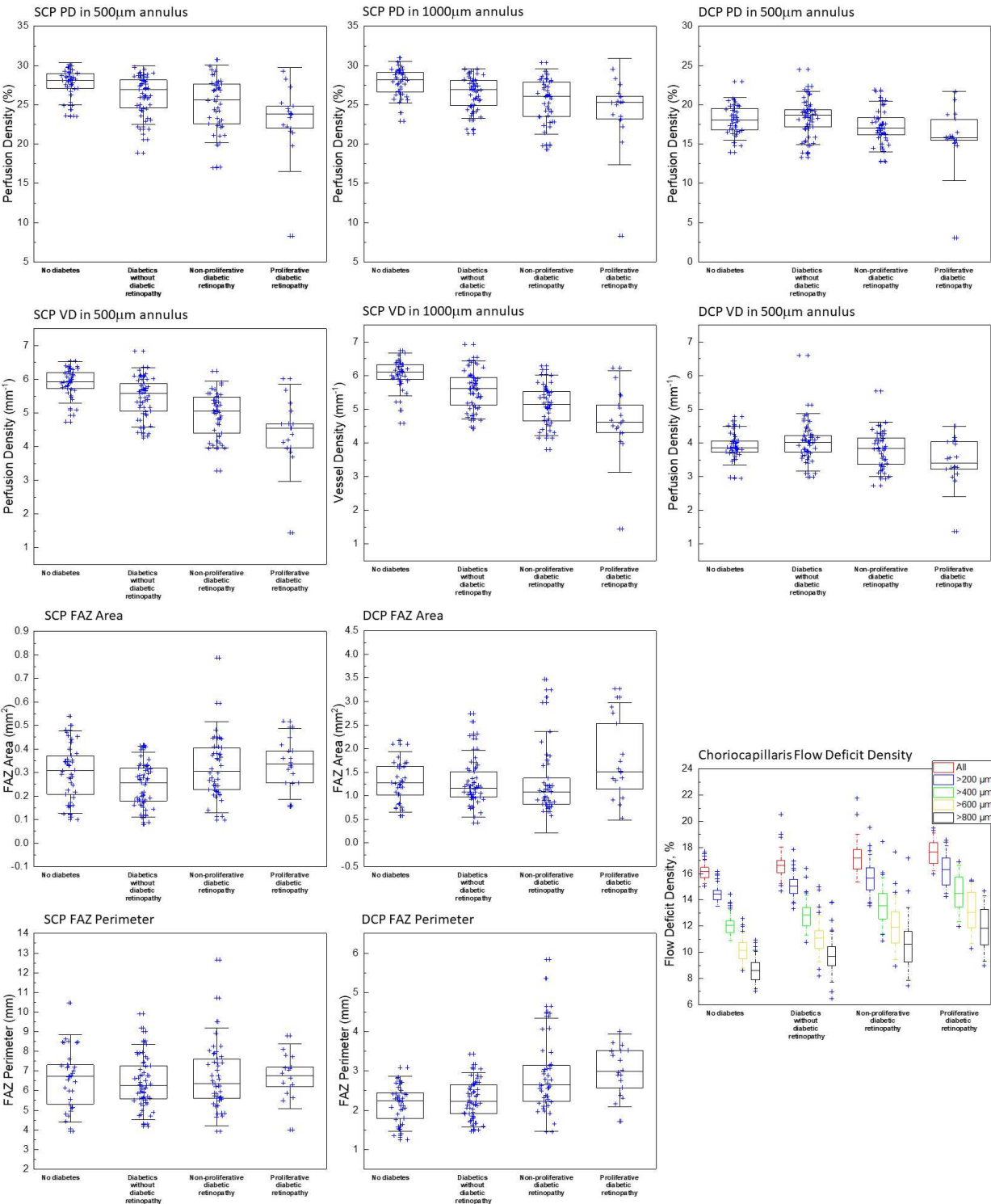

Supplement: Supplementary data [file bjophthalmol-2021-319739supp002.pdf]

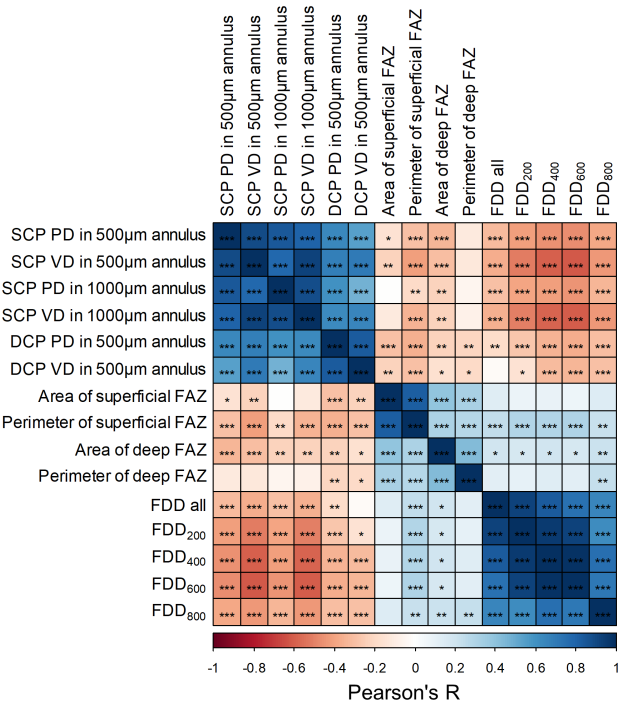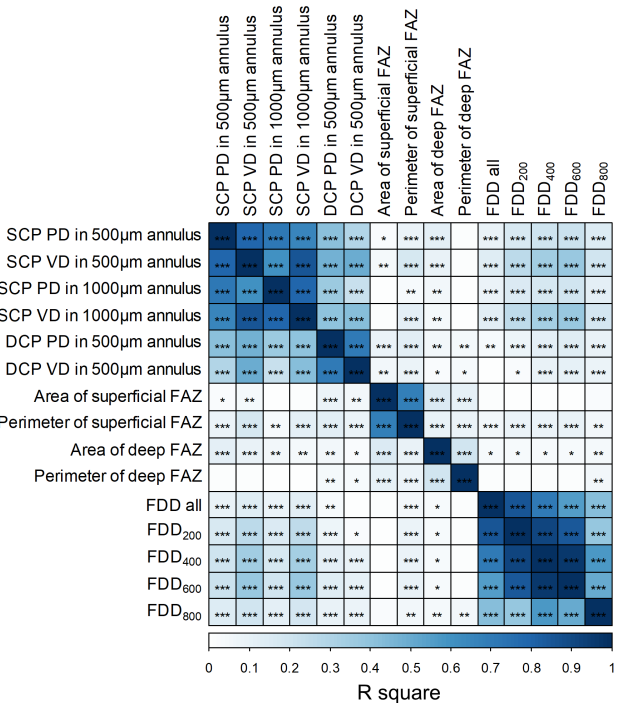

Supplement: Supplementary data [file bjophthalmol-2021-319739supp003.pdf]
